# Hybrid precoding based on matrix-adaptive method for multiuser large-scale antenna arrays

**DOI:** 10.1371/journal.pone.0188723

**Published:** 2017-12-04

**Authors:** Yongpan Feng, Suk Chan Kim

**Affiliations:** Electronics Engineering Department, Pusan National University, Busan, Korea; Kaohsiung Medical University, TAIWAN

## Abstract

Massive multiple-input multiple-output (MIMO) is envisioned to offer a considerable improvement in capacity, but it has a high cost and the radio frequency (RF) chain components have a high power consumption at high frequency. To address this problem, a hybrid analog and digital precoding scheme has been studied recently, which restricts the number of RF chains to far less than the number of antenna elements. In this paper, we consider the downlink communication of a massive multiuser multiple-input single-output (MU-MISO) system and propose an iterative hybrid precoding algorithm to approach the capacity performance of the traditional full digital precoding scheme. We aim to attain a large baseband gain by zero-forcing (ZF) digital precoding on the equivalent channel and then minimize the total power to obtain the optimal RF precoder. Simulation results show that the proposed method can approach the performance of the conventional fully digital precoding with a low computational complexity.

## Introduction

In large-scale multiple-input multiple-output (MIMO) systems, the high capacity performance is rendered by a large number of antennas with a simplified transmit and receive precoding design [1–3]. In traditional precoding, the complex transmit signals can be modified on their modulus and phases at the baseband and then carried through radio frequency (RF) chains to each transmit antennas; thus, each antenna needs to be supported by a dedicated RF chain. In other words, it is necessary that the number of the RF chains is equal to the number of the antenna elements. This is, in fact, unacceptable for massive MIMO systems owing to their high implementation cost and energy consumption.

To address the challenge of a limited number of RF chains, a two-stage hybrid architecture has been studied, in which the analog RF precoding provides the high-dimensional phase-only processing while the digital precoding processes the signal streams at a very low dimension [4–9]. Reference [4] proposed a low-complexity RF precoder by extracting the conjugate transpose of the channel phase array (CTCPA), which is a very simple and useful method. However, its performance is far from the limiting performance of fully digital precoding, especially as the number of antennas and signal-to-noise rate ratio (SNR) increase. An iterative algorithm was proposed in [5], which rewrites the expression for the spectrum efficiency to extract the contribution of each entry of the RF matrix to the objective function. Nevertheless, the complexity of this algorithm is too high. Reference [6] proposed a successive interference cancelation (SIC) based hybrid precoding from the perspective of the energy efficiency. The works in [7–9] developed several iterative hybrid beamforming algorithms based on partial channel knowledge and a variant of matching pursuit method. However, these algorithms are only limited to a certain channel model, i.e., the Saleh-Valenzuela model, which is difficult to apply on other microwave channel models, i.e., doubly dispersive channel model.

In this paper, a multiuser multiple-input single-output (MU-MISO) downlink communication system is considered and we propose an efficient way to design a hybrid precoder, in which an alternating direction method is applied: first, fix the RF precoder and try to seek the optimal digital precoder; second, fix the digital precoder obtained from the first step and try to find the optimal RF precoder; and then, iterate these two steps until convergence. The digital precoder is obtained by the classical ZF method and waterfilling algorithm while the RF precoder is obtained by a fast iterative algorithm based on the normalized matrix adaptive method (NMAM). Desirable performance is demonstrated by the numerical simulation.

## System model

**Fig 1:**
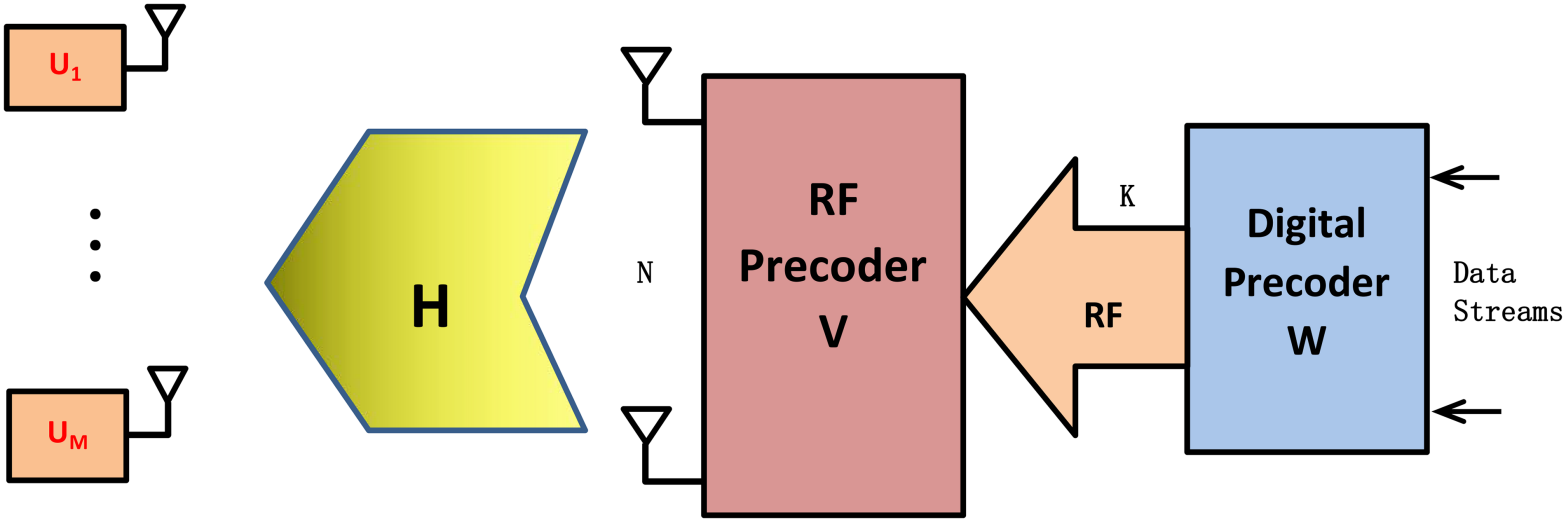
Block diagram of a MU-MISO system with the hybrid precoding architecture at the BS.

Consider a narrowband downlink MU-MISO system as shown in Fig 1, where the base station (BS) is equipped with *N* antennas and *K*(≪ *N*) transmit RF chains, and each of the *M* users is equipped with only one antenna. The transmitted signals first propagate through the low-dimensional digital precoder $W\in C^{K\times S}$ and then pass through the high-dimensional RF precoder $V\in C^{N\times K}$. It should be noted that the RF precoding array is constructed by the phase-shifter array, which means that the modulus of each entry of the RF precoder is equal to a constant number, i.e., |*V*_*n*,*m*_| = 1.

In this paper, we focus on hybrid precoding for the case in which the number of RF chains and the number of data streams *S* both equal to the number of the users, i.e., *S* = *K* = *M*. Then, the received signal stream for the *m*th user can be expressed as

$$y_{m}=h_{m}^{T}{\text{Vw}}_{m}s_{m}+h_{m}^{T}\sum_{l\neq m}{\text{Vw}}_{l}s_{l}+z_{m},$$ (1)

where *s*_*l*_ is the data stream for the *l*th user, $h_{m}^{T}\in C^{1\times N}$ is the complex channel from the BS to the *m*th user, **w**_*l*_ denotes the *l*th column of the digital precoder **W** and $z_{m}∼\text{CN}\left(0,\sigma^{2}\right)$ denotes additive white Gaussian noise.

For such a system with hybrid precoding architecture at the BS, by assuming that $E\left[{\text{ss}}^{H}\right]=I_{M}$, where **s** = [*s*_1_,⋯,*s*_*M*_]^*T*^, the rate expression for the *m*th user can be expressed as

$$R_{m}={\text{log}}_{2}(1+\frac{|h_{m}^{T}{\text{Vw}}_{m}{|}^{2}}{\sigma^{2}+\sum_{l\neq m}{\left|h_{m}^{T}{\text{Vw}}_{l}\right|}^{2}})$$ (2)

## Hybrid precoder design

The problem of interest is to maximize the overall spectral efficiency under total transmit power constraint, assuming perfect knowledge of **h**_*m*_, i.e., we aim to find the optimal hybrid precoders at BS by solving the following problem:

$$\begin{array}{c} \begin{array}{cc} \underset{V,W}{\text{max}}\; & R=\sum_{m=1}^{M}R_{m}\\ s.t.\; & tr({\text{VWW}}^{H}V^{H})\leq P\\ & |V_{n,m}|=1,\forall n,m \end{array} \end{array}$$ (3)

where *P* is the total power budget at the BS and *V*_*n*,*m*_ is the (*n*,*m*)th entry of **V**.

To solve this problem, we utilize the alternating direction method, which iteratively optimizes the RF precoder and digital precoder until convergence. First, we seek to design the digital precoders, assuming that the optimal RF precoder is applied. Then, for the already designed digital precoder, we seek to design the RF precoder.

### Digital precoder design

For a fixed RF precoder **V**, which can be initialized by a random feasible matrix, we consider the product of the channel matrix and the RF precoder as the effective channel, i.e., $\tilde{H}=\text{HV}$, where **H** = [**h**_1_,⋯,**h**_M_]^*T*^.

Considering ZF precoding with power allocation, the solution can be easily found as

$$W={\tilde{H}}^{†}\Lambda^{1/2}=V^{H}H^{H}{\left({\text{HVV}}^{H}H^{H}\right)}^{-1}\Lambda^{1/2}$$ (4)

where (⋅)^†^ is pseudo-inverse operator and **Λ** = diag(λ_1_, ⋯, λ_M_) with λ_*m*_ denoting the received power at the *m*th user.

We note that the only variables in (4) are the received powers, [λ_1_,⋯,λ_*M*_]. By the properties of ZF precoding, we have $|h_{m}^{T}{\text{Vw}}_{l}|=\sqrt{\lambda_{m}}$ at *l* = *m* and 0 elsewhere.

Then, the problem (3) can be rewritten as

$$\begin{array}{c} \begin{array}{cc} \underset{\Lambda }{\text{max}}\; & {\text{log}}_{2}\left|I_{M}+\frac{1}{\sigma^{2}}\Lambda \right|\\ s.t.\; & tr(W^{H}V^{H}\text{VW})\leq P \end{array} \end{array}$$ (5)

The optimal solution of (5) has been found by the waterfilling algorithm in [5].

### RF precoder design

After consider the ZF digital precoding in previous subsection, we now seek to design the RF precoder. Note that the achievable sum rate with ZF precoding depends on the RF precoder **V** only through the power constraint. Therefore, the RF precoder can be captured by the following optimization problem [5]

$$\begin{array}{c} \begin{array}{cc} \underset{V}{\text{min}}\; & tr(W^{H}V^{H}\text{VW})\\ s.t.\; & |V_{n,m}|=1,\forall n,m. \end{array} \end{array}$$ (6)

In order to drop the constraint on the constant modulus for each entry of the RF precoder, we define the RF precoder **V** as a function of its phase matrix,

$$V≜e^{iX}$$ (7)

where $X\in R^{N\times M}$ is the variable matrix and $i=\sqrt{-1}$. The following work focuses on finding a directional matrix $D\in R^{N\times M}$ along which the variable matrix **X** can be updated to obtain a better performance for (6).

For convenience, we define the objective function in (6) as

$$\begin{array}{c} \begin{array}{cc} f(X) & ≜tr(W^{H}V^{H}\text{VW})\\ & =tr[{\left({\text{HVV}}^{H}H^{H}\right)}^{-1}{\text{HVV}}^{H}H^{H}{\left({\text{HVV}}^{H}H^{H}\right)}^{-1}\Lambda ]\\ & =tr[V^{H}V{\left(V^{H}\text{RV}\right)}^{-1}] \end{array} \end{array}$$ (8)

where **R** = **H**^**H**^ **Λ**^−1^ **H**.

We first obtain the derivative of *f*(**X**) with respect to **V** as follows (S1 Appendix):

$$\begin{array}{c} \begin{array}{cc} G & ≜\frac{\partial f(X)}{\partial V}\\ & =\frac{\partial tr[V^{H}V{\left(V^{H}\text{RV}\right)}^{-1}]}{\partial V}\\ & ={\left({\left(V^{H}\text{RV}\right)}^{-1}V^{H}\left(I_{N}-V{\left(V^{H}\text{RV}\right)}^{-1}V^{H}R\right)\right)}^{T} \end{array} \end{array}$$ (9)

where ℜ(⋅) and ℑ(⋅) are the real and imaginary parts respectively.

Then, according to the derivative chain rule for complex number, we obtain the derivative of the objective function with respect to each entry of the variable matrix **X**, which can be expressed as follows

$$\begin{array}{ll} D_{n,m} & =\frac{\partial f\left(X\right)}{\partial X_{n,m}}\\ & =tr\left({\left(\frac{\partial f\left(X\right)}{\partial V}\right)}^{T}\frac{\partial V}{\partial V_{n,m}}\right)\frac{\partial V_{n,m}}{\partial X_{n,m}}\\ & =tr\left(G^{T}P_{n,m}\right)iV_{n,m}\\ & =G_{n,m}iV_{n,m} \end{array}$$ (10)

where $P_{n,m}\in R^{N\times M}$ is a single-entry matrix–1 at (*n*,*m*)th and zero elsewhere, *A*_*n*,*m*_ is the (n,m)th entry of the matrix **A**, and (⋅)^*T*^ is the transpose operator.

So, the directional matrix **D** can be obtained easily as follows,

D=iG∘V, (11)

where ∘ is Hadamard (elementwise) product.

Until now, we obtain the directional matrix **D** for the variable matrix **X**. In order to maintain the updated **X** as a real matrix always, we only take the real part of the directional matrix. Meanwhile, in order to make each step size not too large, we normalize the directional matrix by its 2-norm value. Then, the update equation for the variable matrix can be formulated as follows:

$$X_{t+1}=X_{t}-\delta_{t}\frac{ℜD_{t}}{∥D_{t}{∥}_{F}^{2}},$$ (12)

where *δ*_*t*_ is the *t*-th step size, which can be obtained by the revised Armijo line search method (see Algorithm 1).

The initialization of **X**_0_ can be a random real matrix and the stop time for the update can be when the iteration time is greater than the threshold time or when the 2-norm of the directional matrix is sufficiently small, which can be expressed as *t* > *T*_*threshold*_ or $∥D_{t}{∥}_{F}^{2}<\varepsilon$.

In brief, the design of RF precoder **V** is shown in Algorithm 1.

**Algorithm 1** Design of RF precoder **V**

**Require:** **R**, *δ*_0_, *β*

1: **Initialze:** $X_{0}\in R^{N\times M}$ is a random matrix

2: **while** *t* ≤ *T*_*threshold*_ and $∥D_{t}{∥}_{F}^{2}\geq \varepsilon$ **do**

3:  Calculate $V_{t}=e^{iX_{t}}$, $A_{t}={\left(V_{t}^{H}{\text{RV}}_{t}\right)}^{-1}V_{t}^{H}$.

4:  Calculate **D**_*t*_ = *i*(**A**_**t**_(**I**_**N**_ − **V**_**t**_ **A**_**t**_ **R**))^*T*^ ∘ **V**_*t*_.

5:  Initialze *δ*_*t*_ = *δ*_0_.

6:  // Find the optimal step size.

7:  $F=f\left(X_{t}\right),F_{1}=f\left(X_{t}+\delta_{t}\frac{ℜD_{t}}{∥D_{t}{∥}_{F}^{2}}\right)$

8:  **while** *F* > *F*_1_

9:   *δ*_*t*_ = *βδ*_*t*_

10:    *F* = *F*_1_,

11:    $F_{1}=f\left(X_{t}+\delta_{t}\frac{ℜD_{t}}{∥D_{t}{∥}_{F}^{2}}\right)$

12:  **end while**

13:  Update $X_{t+1}=X_{t}-\delta_{t}\frac{ℜD_{t}}{∥D_{t}{∥}_{F}^{2}}$.

14: **end while**

where *δ*_0_ is a small feasible step size and *β* is lightly greater than 1, i.e., *β* = 1.1.

## Simulation

We numerically compare our proposed NMAM precoding scheme with the Foad’s iterative algorithm in [5] and the CTCPA precoding scheme in [4], which considers the Hermitian transpose of the channel phase array as the RF precoding, i.e., $V_{n,m}^{\text{CTCPA}}=H_{m,n}^{*}/\left|H_{m,n}\right|$. As a reference for the limiting performance, we also simulate the scheme of fully digital precoding, in which every antenna has a dedicated RF chain. In the simulation, the propagation environment between the BS and the user is modeled as a Rayleigh channel (other microwave channel model can be simulated by the same way), in which each element of **H** is an independent and identically distributed (i.i.d) complex Gaussian random variable with unit variance and zero mean, i.e., $H_{m,n}∼\text{CN}\left(0,1\right)$. Meanwhile, we set *δ*_0_ = 1, *β* = 1.1, *T*_*threshold*_ = 100 and *ε* = 0.01.

### Performance analysis

The performances of different schemes under three different pairs of the antennas numbers and the users numbers, i.e., (*M*,*N*) = (8,64), (24,64) and (64,128), were simulated. Meanwhile, we set *S* = *K* = *M* and *δ*_0_ = 1.

**Fig 2:**
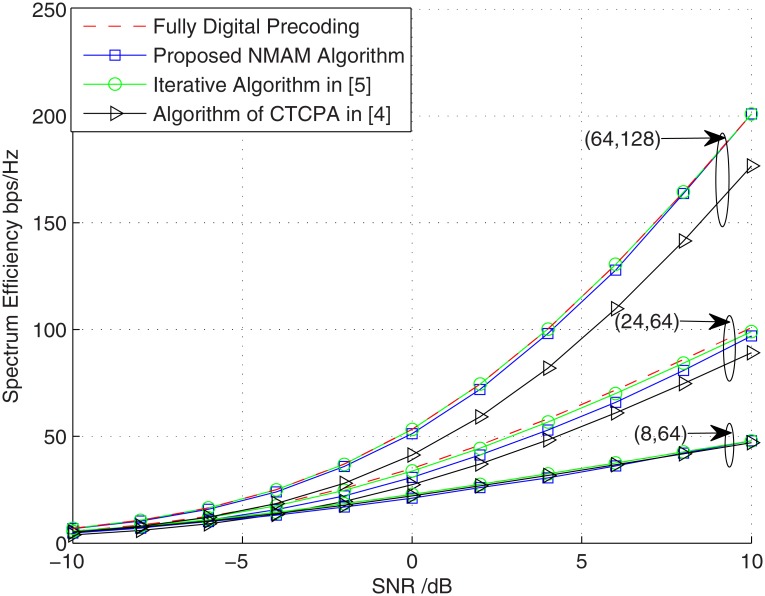
The comparisons among different schemes under different numbers of antennas and users.

The results in Fig 2 show that the proposed method and the algorithm in [5] approach the limiting performance of fully digital precoding, while the CTCPA scheme is becoming difficult to match the performance of fully digital precoding as the numbers of receivers increase. Moreover, it is clear to see in the case of (64,128) that the gap between the performance of fully digital precoding and that of the CTCPA method is becoming large as the SNR increases, while the proposed method and the algorithm in [5] always match the performance of fully digital precoding. This indicates that the proposed algorithm is nearly optimal.

### Quantized RF phase analysis

In practical implementation, the phase of each element in RF precoder is tend to be quantized. Therefore, we need to study the performance of the proposed RF precoder in this realistic scenario. The phase of each entry in RF precoder **V** can be chosen by the following equation,

$$\begin{array}{c} \hat{\phi }=\arg\underset{\phi_{n}}{\text{min}}\left|\phi -\phi_{n}\right|,\;n\in \left[0,1,\cdots ,2^{B}-1\right] \end{array}$$ (13)

where *ϕ* is the unquantized phase and *ϕ*_*n*_ = 2*πn*/2^*B*^.

**Fig 3:**
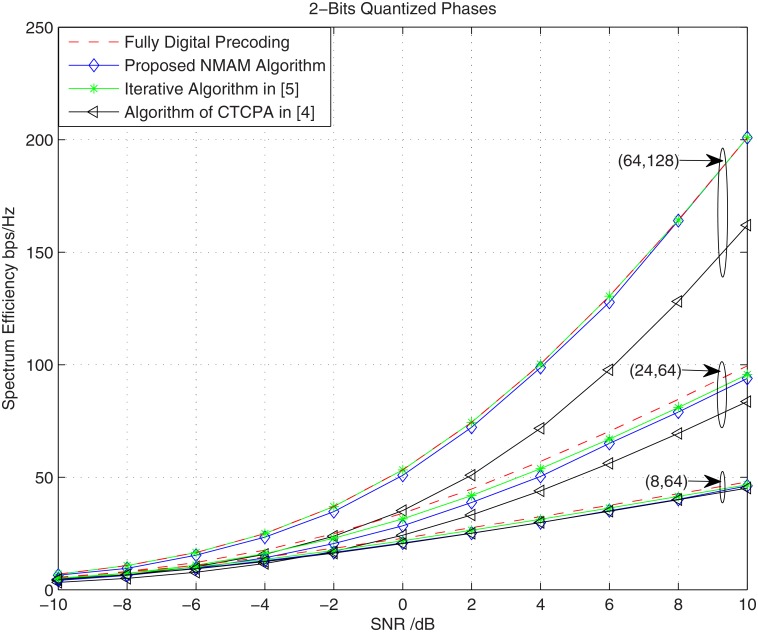
The comparisons among different schemes with 2-bit quantized phase-shifter array.

In this paper, we set *B* = 2, which is a common value in realistic implementation, and the simulation results is shown in Fig 3 as follows,

Simulation results shows that the 2-bit quantized phase greatly preserves the near-optimal performance of the unquantized phase with the proposed method and the algorithm in [5], while the performance of the algorithm in [4] with quantized phase becomes much worse compare with its algorithm with unquantized phase. Therefore, the proposed algorithm can work well in practical implementation.

### Computational complexity analysis

Assume that arithmetic with individual elements has complexity O(1), and we ignore the operations of addition and subtraction. The computational complexity of the matrix multiplication with one *n* × *m* matrix and one *m* × *p* matrix is O(nmp); the computational complexity of the *n* × *n* matrix inversion is $O(n^{3})$ (assume we adopt the Gauss-Jordan elimination method). Then the comparison of computational complexity between the proposed algorithm and the algorithm in [5] is shown in Table 1.

**Table 1:** Computational analysis.

Algorithms	Computational Complexities
Proposed Algorithm	$O(N^{3}+3MN^{2}+2M^{2}N+M^{3})$
Algorithm in [5]	$O(4M^{2}N^{2}+2M^{3}N+M^{4})$

Table 1 clearly shows that the computational complexity of the algorithm in [5] is at least *M* times higher than the proposed algorithm; while the performances of these algorithms are nearly the same. Thus, the efficiency of the proposed algorithm is at least *M* times higher than that of the algorithm in [5].

## Conclusion

In this paper, we considered a hybrid precoding architecture for the MU-MISO downlink wireless communication systems with large-scale antenna arrays. By deducing the derivative of the overall spectral efficiency with respect to the phase matrix of the RF precoder, we proposed an efficient adaptive algorithm of NMAM for designing the RF precoder. We obtain the large baseband gain by ZF digital precoding on the equivalent channel; then, the NMAM is carried out to approach the least power consumption for the transmitter through phased-only RF precoding. Numerical results demonstrated the performance of the proposed algorithm achieves the capacity performance of conventional fully digital precoding processing and has a low computational complexity.

## Supporting information

S1 Appendix Proof of Eq (9). (PDF)
