# Supplementary material for: Hybrid precoding based on matrix-adaptive method for multiuser large-scale antenna arrays
Source: PLoS One. 2017 Dec 4;12(12):e0188723. doi: 10.1371/journal.pone.0188723 (PMC5714346; doi:10.1371/journal.pone.0188723)
Supplement: S1 Appendix — (PDF) [file pone.0188723.s001.pdf]

$$
\begin{aligned}
\partial f(\mathbf{X}) &= \partial tr[\mathbf{V^H V (V^H R V)^{-1}}] \\
&= tr[\partial(\mathbf{V}^H\mathbf{V}(\mathbf{V}^H\mathbf{R}\mathbf{V})^{-1})] \\
&= tr[(\partial\mathbf{V}^H)\mathbf{V}(\mathbf{V}^H\mathbf{R}\mathbf{V})^{-1} \\
&\quad + \mathbf{V}^H(\partial\mathbf{V})(\mathbf{V}^H\mathbf{R}\mathbf{V})^{-1} + \mathbf{V}^H\mathbf{V}\partial(\mathbf{V}^H\mathbf{R}\mathbf{V})^{-1}] \\
&= tr[(\partial\mathbf{V}^H)\mathbf{V}(\mathbf{V}^H\mathbf{R}\mathbf{V})^{-1} + \mathbf{V}^H(\partial\mathbf{V})(\mathbf{V}^H\mathbf{R}\mathbf{V})^{-1} \\
&\quad - \mathbf{V}^H\mathbf{V}(\mathbf{V}^H\mathbf{R}\mathbf{V})^{-1}((\partial\mathbf{V}^H)\mathbf{R}\mathbf{V} \\
&\quad + \mathbf{V}^H\mathbf{R}\partial\mathbf{V})(\mathbf{V}^H\mathbf{R}\mathbf{V})^{-1}]
\end{aligned} \tag{1}
$$

First, the derivative is found with respect to the real part of $\mathbf{V}$

$$
\begin{aligned}
\frac{\partial f(\mathbf{X})}{\partial\Re\mathbf{V}} &= \frac{tr[(\partial\mathbf{V}^H)\mathbf{V}(\mathbf{V}^H\mathbf{R}\mathbf{V})^{-1}]}{\partial\Re\mathbf{V}} \\
&\quad + \frac{tr[\mathbf{V}^H(\partial\mathbf{V})(\mathbf{V}^H\mathbf{R}\mathbf{V})^{-1}]}{\partial\Re\mathbf{V}} \\
&\quad - \frac{\mathbf{V}^H\mathbf{V}(\mathbf{V}^H\mathbf{R}\mathbf{V})^{-1}(\partial\mathbf{V}^H)\mathbf{R}\mathbf{V}}{\partial\Re\mathbf{V}} \\
&\quad - \frac{\mathbf{V}^H\mathbf{V}(\mathbf{V}^H\mathbf{R}\mathbf{V})^{-1}\mathbf{V}^H\mathbf{R}\partial\mathbf{V}(\mathbf{V}^H\mathbf{R}\mathbf{V})^{-1}}{\partial\Re\mathbf{V}} \\
&= \mathbf{V}(\mathbf{V}^H\mathbf{R}\mathbf{V})^{-1} + ((\mathbf{V}^H\mathbf{R}\mathbf{V})^{-1}\mathbf{V}^H)^T \\
&\quad - \mathbf{R}\mathbf{V}\mathbf{V}^H\mathbf{V}(\mathbf{V}^H\mathbf{R}\mathbf{V})^{-1} \\
&\quad - ((\mathbf{V}^H\mathbf{R}\mathbf{V})^{-1}\mathbf{V}^H\mathbf{V}(\mathbf{V}^H\mathbf{R}\mathbf{V})^{-1}\mathbf{V}^H\mathbf{R})^T
\end{aligned} \tag{2}
$$

In addition, the derivative is found with respect to the imaginary part of $\mathbf{V}$

$$
\begin{aligned}
i\frac{\partial f(\mathbf{X})}{\partial\Im\mathbf{V}} &= i\frac{tr[(\partial\mathbf{V}^H)\mathbf{V}(\mathbf{V}^H\mathbf{R}\mathbf{V})^{-1}]}{\partial\Im\mathbf{V}} \\
&\quad + i\frac{tr[\mathbf{V}^H(\partial\mathbf{V})(\mathbf{V}^H\mathbf{R}\mathbf{V})^{-1}]}{\partial\Im\mathbf{V}} \\
&\quad - i\frac{\mathbf{V}^H\mathbf{V}(\mathbf{V}^H\mathbf{R}\mathbf{V})^{-1}(\partial\mathbf{V}^H)\mathbf{R}\mathbf{V}}{\partial\Im\mathbf{V}} \\
&\quad - i\frac{\mathbf{V}^H\mathbf{V}(\mathbf{V}^H\mathbf{R}\mathbf{V})^{-1}\mathbf{V}^H\mathbf{R}\partial\mathbf{V}(\mathbf{V}^H\mathbf{R}\mathbf{V})^{-1}}{\partial\Im\mathbf{V}} \\
&= \mathbf{V}(\mathbf{V}^H\mathbf{R}\mathbf{V})^{-1} - ((\mathbf{V}^H\mathbf{R}\mathbf{V})^{-1}\mathbf{V}^H)^T \\
&\quad - \mathbf{R}\mathbf{V}\mathbf{V}^H\mathbf{V}(\mathbf{V}^H\mathbf{R}\mathbf{V})^{-1} \\
&\quad + ((\mathbf{V}^H\mathbf{R}\mathbf{V})^{-1}\mathbf{V}^H\mathbf{V}(\mathbf{V}^H\mathbf{R}\mathbf{V})^{-1}\mathbf{V}^H\mathbf{R})^T
\end{aligned} \tag{3}
$$

Hence, derivative yields

$$
\begin{aligned}
\frac{\partial f(\mathbf{X})}{\partial\mathbf{V}} &= \frac{1}{2}\Big(\frac{\partial f(\mathbf{X})}{\partial\Re\mathbf{V}} - i\frac{\partial f(\mathbf{X})}{\partial\Im\mathbf{V}}\Big) \\
&= ((\mathbf{V^H R V})^{\mathbf{-1}}\mathbf{V^H}(\mathbf{I_N} - \mathbf{V}(\mathbf{V^H R V})^{\mathbf{-1}}\mathbf{V^H R}))^T
\end{aligned} \tag{4}
$$
